# Detection of *Alicyclobacillus* spp. in Fruit Juice by Combination of Immunomagnetic Separation and a SYBR Green I Real-Time PCR Assay

**DOI:** 10.1371/journal.pone.0141049

**Published:** 2015-10-21

**Authors:** Rui Cai, Zhouli Wang, Yahong Yuan, Bin Liu, Ling Wang, Tianli Yue

**Affiliations:** College of Food Science and Engineering, Northwest A&F University, Yangling, Shaanxi, 712100, China; University of Campinas, BRAZIL

## Abstract

An approach based on immunomagnetic separation (IMS) and SYBR Green I real-time PCR (real-time PCR) with species-specific primers and melting curve analysis was proposed as a rapid and effective method for detecting *Alicyclobacillus* spp. in fruit juices. Specific primers targeting the 16S rDNA sequences of *Alicyclobacillus* spp. were designed and then confirmed by the amplification of DNA extracted from standard strains and isolates. Spiked samples containing known amounts of target bacteria were used to obtain standard curves; the correlation coefficient was greater than 0.986 and the real-time PCR amplification efficiencies were 98.9%- 101.8%. The detection limit of the testing system was 2.8×10^1^ CFU/mL. The coefficient of variation for intra-assay and inter-assay variability were all within the acceptable limit of 5%. Besides, the performance of the IMS-real-time PCR assay was further investigated by detecting naturally contaminated kiwi fruit juice; the sensitivity, specificity and accuracy were 91.7%, 95.9% and 95.3%, respectively. The established IMS-real-time PCR procedure provides a new method for identification and quantitative detection of *Alicyclobacillus* spp. in fruit juice.

## Introduction

*Alicyclobacillus* spp. are one of the most important quality and safety factors in acidic food products [1, 2]. The pasteurization processes are not effective against the thermo-acidophilic spore-forming microorganisms and their spores can survive conventional heat processes. Growth of *Alicyclobacillus* spp. in fruit juices can cause sour spoilage [3–5]. The metabolic products of these bacteria include guaiacol, dihalophenol and dibromophenol, which result in undesirable medicinal or antiseptic off-odors [6, 7]. At present, *Alicyclobacillus* spp. have been isolated from apple, kiwi, grapefruit, orange and many kinks of fruit products and have posed a major challenge to the fruit juice and beverage industry [8–11]. The development of species-specific methods for the detection and identification of *Alicyclobacillus* spp. in fruit juice is critical.

The traditional culture methods for target bacteria detection are labor-intensive, time-consuming and inefficient, so they cannot guarantee the absence of *Alicyclobacillus* spp. in fruit juice [12–14]. As a sensitive and rapid method, the polymerase chain reaction (PCR) has the potential to overcome these difficulties [15–17]. SYBR Green I based real-time PCR (real-time PCR), which has been developed for detection and quantification of microorganisms in many samples, demonstrates advantages in simplifying primer design, lowering test cost and standardizing experimental procedures [18–21]. And yet for all that, the complex background interference and the non-target flora have inhibited the direct application of PCR-based detection methods to food samples [3, 22, 23]. The removal of inhibitory substances is a major step in preparation of samples for PCR-based detection of target bacteria. As a time-saving and high specificity method, immunomagnetic separation (IMS) has been evaluated as an efficient pre-treatment for the separation and concentration of target organisms [24–26]. In this process, immunomagnetic nanoparticles (IMPs) are obtained by coating the specific antibody onto the surface of magnetic nanoparticles. The target bacteria are captured onto the IMPs and the resultant “bacteria-bead” complexes are removed from the system by the application of a magnetic field. In this way, the target bacteria are removed from food debris and competing microorganisms; cell numbers can be rapidly increased to improve the sensitivity of detection assays and the total analysis time are obviously reduced. For detection, these immunocaptured bacteria are lysed and filtrated, and the supernatant is used as template. At present, the combination of IMS with detection methods such as direct plating, PCR, Enzyme Linked Immunosorbent Assay (ELISA) and others has been used to further increase their detection sensitivity for foodborne pathogens [27, 28].

The aim of this study was to use the IMS test plus a SYBR Green I real-time PCR assay (IMS-real-time PCR) for detection of *Alicyclobacillus* spp. in kiwi fruit juice. The target 16S rDNA sequences were identified and the primers set suitable for the real-time PCR system was developed. The assay for the identification and quantification of *Alicyclobacillus* spp. in fruit juice using IMS-real-time PCR was established. The correlation coefficient, detection limit, repeatability and amplification efficiency were evaluated.

## Materials and Methods

### Bacterial strains

The strains used in this study are listed in Table 1. Twenty standard strains including 15 *Alicyclobacillus* spp. and 5 *Bacillus* spp. were purchased from German Resource Centre for Biological Material (DSMZ). The other twenty-two wild-type isolates were isolated from apple or kiwi fruit orchard or fruit juice production line and obtained from microbiology laboratory of the College of Food Science and Engineering, Northwest A&F University, Yangling, China [8, 29, 30]. All of the strains were stored on the slants at 4°C for routine use.

**Table 1:** Bacteria used for primer verification and specificity testing of the IMS- real-time PCR.

Strain Name	Strain No.	Strain Name	Strain No.
*A*. *acidoterrestris*	DSM-2498 ^a^	*A*. *acidocaldarius*	DSM-446 ^a^
*A*. *acidoterrestris*	DSM-3922 ^a^	*A*. *acidocaldarius*	DSM-448 ^a^
*A*. *acidoterrestris*	DSM-3923 ^a^	*A*. *acidocaldarius*	DSM-449 ^a^
*A*. *acidoterrestris*	DSM-3924 ^a^	*A*. *acidocaldarius*	DSM-451 ^a^
*A*. *acidoterrestris*	C- ZJB-12-01 ^b^	*A*. *pomorum*	DSM-14955 ^a^
*A*. *acidoterrestris*	C- ZJB-12-11 ^b^	*A*. *hesperidum*	DSM-12489 ^a^
*A*. *acidoterrestris*	C-ZJB-12-18 ^b^	*A*. *sendaiensis*	DSM-17614 ^a^
*A*. *acidoterrestris*	C- ZJB-12-23 ^b^	*A*. *fastidiosus*	DSM-17978 ^a^
*A*. *acidoterrestris*	C-ZJB-12-42 ^b^	*A*.*herbarius*	DSM-13609 ^a^
*A*. *acidoterrestris*	LC-4 ^b^	*A*.*herbarius*	C-ZJB-12-47 ^b^
*A*. *acidiphilus*	DSM-14558 ^a^	*A*.*herbarius*	C-ZJB-12-56 ^b^
*A*. *contaminans*	DSM-17975 ^a^	*A*.*herbarius*	C-ZJB-12-85 ^b^
*A*. *contaminans*	C-ZJB-12-67 ^b^	*B*. *subtilis*	DSM-10 ^a^
*A*. *contaminans*	YL-5 ^b^	*B*. *subtilis*	YL-3 ^b^
*B*. *brevis*	DSM-30 ^a^	*B*. *coagulans*	C- ZJB-12-14 ^b^
*B*. *licheniformis*	DSM-13 ^a^	*B*. *coagulans*	C- ZJB-12-15 ^b^
*B*. *megaterium*	DSM-32 ^a^	*B*. *fumarioli*	C- ZJB-12-21 ^b^
*B*. *ginsengihumi*	DSM-18134 ^a^	*B*. *fumarioli*	C- ZJB-12-39 ^b^
*B*. *ginsengihumi*	LC-8 ^b^	*B*. *fumarioli*	C- ZJB-12-70 ^b^
*B*. *ginsengihumi*	C- ZJB-12-22 ^b^	*B*. *fumarioli*	C- ZJB-12-83 ^b^
*B*. *ginsengihumi*	C- ZJB-12-82 ^b^	*B*. *fumarioli*	C- ZJB-12-84 ^b^

Various superscripts in lowercase indicate the sources of strains:

^a^ The strain was purchased from German Collection of Microorganisms and Cell Cultures (DSMZ);

^b^ The strains were obtained from microbiology laboratory of the College of Food Science and Engineering, Northwest A&F University (NWSUAF).

### DNA extraction

The extraction and quantitation of DNA were conducted as our previously reported [14]. Cells were collected from 5 mL of bacterial culture or its dilutions by centrifugation at 6,000 ×g for 10 min at 4°C. Collected cells were treated with 20 mg/mL of lysozyme in enzymatic lysis buffer for 30 min at 37°C. Genomic DNA was extracted using a commercial isolation kit following the manufacturer’s instructions (DNeasy@ Tissue Kit, TaKaRa Biotechnology Co.,Ltd, Dalian, China). DNA was eluted with 100 μL of elution buffer from the same kit. DNA concentrations were measured at 260/280 nm using NanoDrop (ND-2000 Spectrophotometer, Saveen Werner, U.S. state).

### Immunomagnetic separation

For detection, the samples were treated according to our previously developed immunocapture procedure [3, 31]. Five milligrams of IMPs and 2 mL of each sample were added into different tubes followed by cultivation at 37°C for 30 min under stirring (150 rpm). Bacteria-bead complexes were separated by magnetic decantation, washed three times with 0.01 mol/L sodium phosphate buffer (PBS, pH 7.4) and resuspended in 200 μL TE buffer (10mM Tris-HCl, 1mM EDTA, pH = 8.0). Genomic DNA was released by boiling the solution for 10 min. After centrifugation at 6,000 ×g for 5 min (4°C), 100 μL of the supernatant was collected as template. Aliquots (50 μL) of the supernatant was purified by the filter paper (Whatman, General Electric Co., Fairfield,Conn., U.S. state) to remove the PCR inhibitor. The total volume of purified DNA was stored at -20°C and subjected to real-time PCR for detection. As a control, the other part of template was directly used for the real-time PCR amplification.

### Design of primers

For real-time PCR primer design, DNA sequences (16S rDNA) of *A*. *acidoterrestris* (DSM-2498, DSM-3922, DSM-3923, DSM-3924), *A*. *contaminans* (DSM-17975), *A*. *acidocaldarius* (DSM-446, DSM-448, DSM-449, DSM-451), *A*. *fastidiosus* (DSM-17978), *A*. *herbarius* (DSM-13609), *A*. *hesperidum* (DSM-12489), *A*. *sendaiensis* (DSM-17614), *A*. *acidiphilus* (DSM-14558) and *A*. *pomorum* (DSM-14955) were obtained from GenBank (National Center for Biotechnology Information, Bethesda, Maryl., U.S. state). Then these sequences were analyzed and aligned for primers design using software Primer Premier 6 (Premier Biosoft International, Canada). A further computer-based specificity search was conducted by Clone manager suite 7.0 to confirm the specificity of designed primers. In this way, a short amplicon (188 bp) was chosen for real-time PCR analysis aiming to detect *Alicyclobacillus* spp.. Two conserved oligonucleotides (the forward primer 5’-ATGCGTAGATATGTGGAGGA-3’ and the reverse primer 5’- CAGG CGGAGTGCTTATTG-3’) were derived; the melting temperature (*Tm*) was 51.1°C and the length of them were 20 and 18 bases, respectively. After that, the primers were synthesized at TaKaRa Biotechnology Co., Ltd., Dalian, China, and the specificity was confirmed by PCR amplification with the parameters described below.

### Real-time PCR assay

A real-time PCR assay for detection and quantification of *Alicyclobacillus* spp. DNA in biological samples was developed. This procedure was carried out in final reaction volumes of 25 μL with 12.5 μL of SYBR Green I Premix Ex-Taq^TM^ (TaKaRa Biotechnology Co., Ltd., Dalian, China), 0.5 μL of each primer set (10 μM), 1.0 μL of template DNA and 10.5 μL of ddH_2_O. Thermal cycling was performed on the iCycler-iQ5 thermal cycler (BioRad, U.S. State) using the cycling conditions: pre-denaturation at 95°C for 10 min followed by 40 cycles of denaturation at 95°C for 15 s, annealing and extension at 60°C for 1 min. The amplification program was then followed by a melting cycle of 95°C for 0 s, 60°C for 30 s, and slow heating to 95°C for 0 s, with a transition rate of 0.5°C/s. The real-time PCR amplification of the targeted DNA was monitored by the increase of the fluorescence in real time. The positive amplification of 16S rRNA gene was determined when the PCR cycle crossed the threshold cycle (*C*_*T*_) value and confirmed by the DNA melt curve analysis.

### Specificity and sensitivity of real-time PCR assay

The specificity of the real-time PCR was determined by comparing results when different DNA samples of *Alicyclobacillus* spp. and *Bacillus* spp. were used as templates (Table 1). The cultured bacteria were suspended in sterile water with the cell concentration of 10^4^−10^5^ CFU/mL. The DNA of each strain was extracted by isolation kit and used in the real-time PCR reactions. For evaluating the sensitivity of the real-time PCR, *A*. *acidoterrestris* (DSM-3923) with a concentration of 2.8×10^7^ CFU/mL was subjected to a 10-fold serial dilution in sterile water. The extracted DNA was used as template for the real-time PCR amplification. Each dilution was carried out three times in triplicate and operated by real-time PCR.

### Evaluation of the IMS- real-time PCR assay

The artificially contaminated kiwi fruit juice was prepared as the spiked samples and the performance of the developed IMS-real-time PCR assay was evaluated. The culture solution of *A*. *acidoterrestris* (DSM-3923) was diluted tenfold in kiwi fruit juice, with the cell concentrations ranging from 2.8×10^−1^ CFU/mL to 2.8×10^6^ CFU/mL. The bacteria diluted by sterile water were considered as control. Then these prepared samples were immunocaptured by IMPs and detected by the real-time PCR assay. The samples without IMS enrichment were also considered as a control and tested by the same approach. At the same time, the obtained data were analyzed statistically and the reproducibility of the results was assessed. Intra-assay and inter-assay variability was determined according to MIQE (Minimal Information for Publication of Quantitative Real time PCR Experiments) guidelines [32].

### Samples collection

A total of 86 natural kiwi fruit juice samples were used to evaluate the effectiveness of the IMS-real-time PCR. Sixty-nine of them was obtained from the kiwi fruit juice production line of Yang ling global horticulture Co., Ltd, and the other 17 samples within the quality guarantee period were purchased from the local supermarkets in Yangling, Shaanxi, China in 2014. For detection, the soluble solid content of samples was adjusted below 15°Brix and then these samples were treated according to our previously developed immunocapture procedure. Then the genomic DNA was obtained and detected by the established real-time PCR. The samples were also detected by a standardized pour plating method issued by the Japan Fruit Juice Association and the results were compared. The sensitivity, specificity and accuracy of the IMS-real-time PCR procedure were also evaluated [33, 34].

### Statistical analysis

Standard curves were obtained for the *A*. *acidoterrestris* (DSM 3923) cultivar following the method described by our previously report [14]. The curves were generated by plotting *C*_*T*_ values as a function of the logarithm of known spiked quantities and determined in triplicate. The correlation coefficient (*R* ^*2*^) was calculated and used to verify the fit of the trend line to the points on the curve. The amplification efficiency (*E*) was estimated by the formula *E* = 10^−1/s^-1, where *s* is the slope of the standard curve [26]. From the standard *C*_*T*_ plots, it was possible to quantify samples with unknown amounts of *Alicyclobacillus* spp.. Each experiment was carried out three times in triplicate.

## Results

### Specificity of the real-time PCR assay

**Fig 1:**
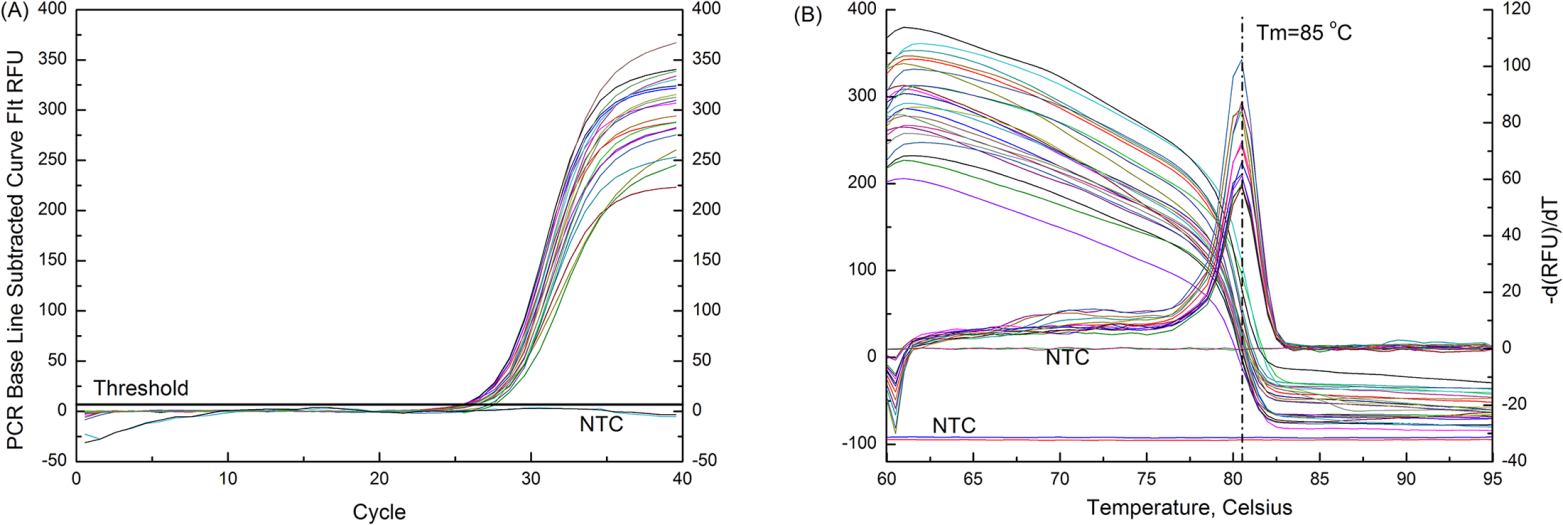
Detection of *Alicyclobacillus* spp. by the proposed real-time PCR assay with the 16S rDNA primer. (A) The representative real-time PCR amplification curves of various *Alicyclobacillus* spp. strains; (B) The melting curve analysis for *Alicyclobacillus* spp. amplicons.

In this study, the primer set was designed and twenty-six *Alicyclobacillus* spp. and sixteen *Bacillus* spp. were used as representative strains to evaluate the specificity of the real-time PCR assay. For detection, the genomic DNA of each strain was extracted as template and the DNA concentration was about 20 ng/μL. The representative real-time PCR amplification curves were presented in Fig 1(A). It was shown that the tested *Alicyclobacillus* spp. were positive in the real-time PCR with the mean *C*_*T*_ values of 26.0 ± 1.0 and the non- *Alicyclobacillus* spp. had no amplification of DNA. To verify the specificity of the reactions using SYBR Green I as a fluorescent dye, melting curve analysis was performed and a reproducible distinct melting point of 80.5°C was observed for all *Alicyclobacillus* spp. amplicons (Fig 1B). The results indicated that the established real-time PCR assay can be used for the detection of *Alicyclobacillus* spp.

### Sensitivity of the real-time PCR assay

**Fig 2:**
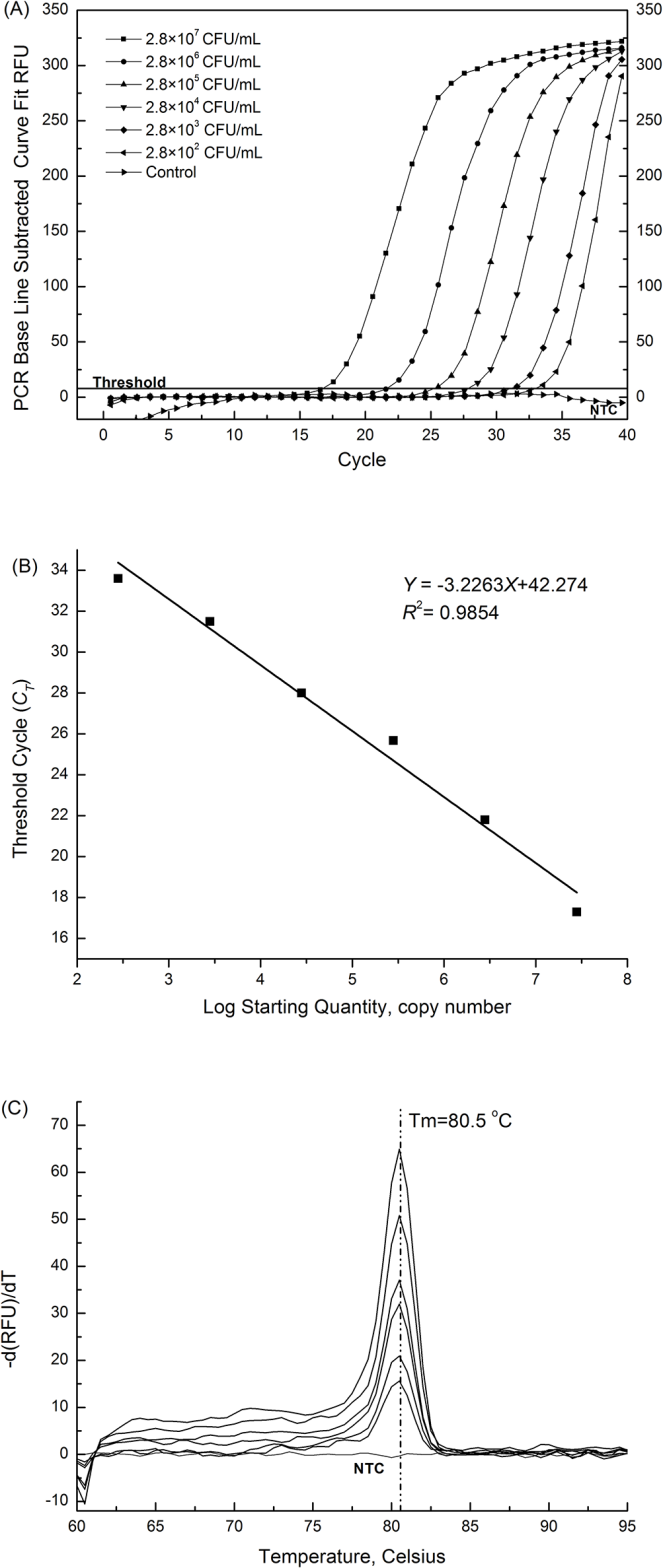
Sensitivity of the real-time PCR detection of *Alicyclobacillus* spp.. (A) Real-time PCR amplification of the target gene fragment with different concentratioans. NTC: no template control; (B) Standard curve generated from the data plotted in panel; (C) Melt peaks of real-time PCR products of 10-fold serial dilutions of *Alicyclobacillus* spp.

The genomic DNA obtained from *A*. *acidoterrestris* (2.8×10^0^ to 2.8 ×10^7^ CFU/ mL) were used as template to evaluate the sensitivity of the developed system. As seen in Fig 2(A), the detect limit of the established real-time PCR assay was about 2.8×10^2^ CFU/mL. The curve showed linear regression relationship with coefficient of determination (*R* ^*2*^) of 0.9854 and a slope of *Y* = −3.2263*X* + 42.274 corresponding to the amplification efficiency of 104.2% (Fig 2B). The results indicated that a high correlation between the cell concentrations and corresponding *C*_*T*_ values. With prudent consideration, samples with *C*_*T*_ value over 35 cycles (corresponding to the concentration of cells less than 10^2^ CFU/mL) were regarded as negative in this study. In addition, only one single sharp peak at 80.5°C was visible in the melt peak chart of 10-fold serial dilutions of tested cells and there was no primer-dimers and non-specific product (Fig 2C). Based on the calculations, the assay was able to amplify 2.8×10^2^ CFU/mL of *Alicyclobacillus* spp.

### The performance of the IMS-real-time PCR assay

To improve the performance of the developed assay, IMS was used for isolation and enrichment of *A*. *acidoterrestris* (DSM-3923) from the artificially contaminated kiwi fruit juice. It was found that the established method allowed the quantification of target strains in the linear dynamic range of 10^1^−10^6^ CFU/mL. The detection limit of the real-time PCR assay was improved one order of magnitude and that was 2.8×10^1^ CFU/mL. The correlation coefficient obtained from the standard curves were greater than 0.986, and the amplification efficiencies of kiwi fruit juice and sterile water samples were 98.9% and 101.8%, respectively. With the pre-treatment of IMS technique, the background interference of the juice ingredients had little influence on detection of *Alicyclobacillus* spp.. The amplification efficiencies and detection limit obtained from fruit juice sample were consistent with the results from sterile water. In addition, the reproducibility of the developed assays was evaluated based on the *C*_*T*_ values obtained from three separate reactions within each run (intra-assay) or in three different runs (inter-assay). For each dilution, the mean, standard deviation (SD) and coefficient of variation (CV) were calculated based on the *C*_*T*_ values (Table 2). The calculated SD and CV values for intra-assay were ranging from 0.08 to 0.30 and from 0.22% to 1.38%, respectively. The SD and CV of inter-assay were slightly higher than that of intra-assay with ranges from 0.44 to 0.70 and from 1.35% to 2.59%, respectively. The CV for intra-assay and inter-assay variability were all within the acceptable limit of 5%, which indicated capability of the assay to generate reproducible results [35]. The results showed that the developed IMS-real-time PCR method exhibited perfect performance for the detection of *Alicyclobacillus* spp. in fruit juice.

**Table 2:** Repeatability of *Ct* values in intra- and inter-assays using the IMS- real-time PCR.

Assay No.	Cycle of threshold (Ct) for *A*. *acidoterrestris* (DSM-3923) detection (CFU/mL)					
	10^6^	10^5^	10^4^	10^3^	10^2^	10^1^
Intra-assay variation						
1	19.17	22.92	26.14	29.37	32.31	35.82
2	19.69	22.32	26.45	29.84	32.65	35.73
3	19.32	22.62	26.53	29.42	32.60	35.66
Ct (means±SD)	35.74±0.08	32.52±0.18	29.54±0.26	26.38±0.21	22.62±0.30	19.39±0.27
CV (%)	0.22	0.55	0.87	0.78	1.33	1.38
Inter-assay variation						
1	19.17	22.92	26.14	29.37	32.31	35.82
2	19.71	22.18	27.53	30.68	32.87	36.29
3	20.04	23.32	26.76	29.89	33.19	35.17
Ct (means±SD)	35.76±0.56	32.79±0.44	29.98±0.66	26.81±0.70	22.81±0.58	19.64±0.44
CV (%)	1.57	1.35	2.20	2.59	2.53	2.24

### Samples detection

In this study, 86 naturally contaminated kiwi fruit juice samples were detected by the IMS-real-time PCR and standardized pour plating method. As shown in Table 3, 11 samples obtained from kiwi fruit production line were identified as positive and the others were all negative by both testing methods. One fruit juice sample identified as *Alicyclobacillus* spp. negative by IMS-real-time PCR was positive using YSG agar. Three samples displayed positive by IMS-real-time PCR, but was *Alicyclobacillus* spp. negative by plating culture method. It showed that the IMS-real-time PCR procedure and pour plating method were consistent for detection of *Alicyclobacillus* spp. in 82 fruit juice samples. The sensitivity, specificity and accuracy of the developed assay were 91.7%, 95.9% and 95.3%, respectively. The results indicated that the proposed IMS-real-time PCR could be effectively used for the detection of *Alicyclobacillus* spp. in fruit juice.

**Table 3:** Comparison of the IMS-real-time PCR and the standardized pour plate method in naturally contaminated kiwi fruit juice products detection.

YSG agar	IMS-real-time PCR		Total	Sensitivity ^a^ (100%)	Specificity ^b^ (100%)	Accuracy ^c^ (100%)
	Positive	Negative				
**Positive**	11	1	12	91.7%	95.9%	95.3%
**Negative**	3	71	74			
**Total**	14	72	86			

^a^ Sensitivity = TP/(TP+FN) ×100% (TP: True positive, FN: False negative);

^b^ Specificity = TN/(TN+FP) ×100% (TN: True negative, FP: False positive);

^c^ Accuracy = (TP+TN)/(TP+TN+FP+FN) ×100%

## Discussion

*Alicyclobacillus* spp. are among the most important spoilage bacteria in fruit juice. Since they were first isolated in 1984, *Alicyclobacillus* spp. have become a major concern to the global juice and beverage industries [1, 4, 9]. Currently, the culture-based techniques are mainly used as routine analysis of fruit juice products in world famous enterprises [2, 9]. The development of rapid and reliable technique for the detection of *Alicyclobacillus* spp. in fruit juice and beverage is urgently needed.

With the advantages of less expensive, simple in primer design and universal real-time-PCR protocols suitable for target sequences, SYBR Green I based real-time PCR can be applied directly to any gene without the need to design and synthesize fluorescently labeled target specific probes [19, 21, 36]. In this study, specific primers based on the 16S rDNA sequences of fifteen representative strains were designed using software Primer Premier 6, evaluated by Clone manager suite 7.0 and confirmed by the amplification of DNA extracted from different standard strains and isolates. A real-time PCR assay was developed and then combined with IMS for detecting *Alicyclobacillus* spp. in fruit juice.

As far as we know, this is the first report on the detection of *Alicyclobacillus* spp. by IMS-real-time PCR. In our previous study, an immunocapture procedure has been established for separation and enrichment of *Alicyclobacillus* spp.. The ELISA or PCR was combined with IMS to shorten the total analysis time and improve the sensitivity for detection of *Alicyclobacillus* spp.. These methods contribute to the qualitative detection and differentiation of target strains in an efficient way [3, 33]. In this study, an effective and reliable quantitative testing method of IMS-real-time PCR was provided for accurate identification of *Alicyclobacillus* spp.. The correlation coefficient of the standard curves was greater than 0.986 and the real-time PCR amplification efficiencies were 98.9%-101.8%. The detection limit of the IMS-real-time PCR assay was 2.8×10^1^ CFU/mL. The sensitivity, specificity and accuracy of the testing system were 91.7%, 95.9% and 95.3%, respectively. The results showed that the IMS-real-time PCR procedure could be potentially useful for detecting *Alicyclobacillus* spp. in fruit products.

In conclusion, a rapid and sensitive IMS-real-time PCR assay was developed for detection of *Alicyclobacillus* spp. in kiwi fruit juice. The established method allowed the quantification of target strains in the linear dynamic range of 10^1^−10^6^ CFU/mL with high correlation and PCR efficiency. Due to its high throughput nature, this approach is convenient for identification of target strains from different fruit juice samples. Further, this assay can be considered as an alternative to the regular methods of *Alicyclobacillus* spp. detection such as plating culture, conventional PCR and ELISA.
