# Correction: Detection of *Alicyclobacillus* spp. in Fruit Juice by Combination of Immunomagnetic Separation and a SYBR Green I Real-Time PCR Assay

**DOI:** 10.1371/journal.pone.0146556

**Published:** 2016-01-04

**Authors:** Rui Cai, Zhouli Wang, Yahong Yuan, Bin Liu, Ling Wang, Tianli Yue

The second author Dr. Zhouli Wang is not a corresponding author. The corresponding author is Dr. Tianli Yue (yuetl305@nwsuaf.edu.cn).
